# Supplementary material for: Determination of the Assigned Values of Blood Cells by an Impedance Method for Hematological Reference Samples Used in Hematology External Quality Assessment (EQA) Programs
Source: Biomedicines. 2022 Dec 7;10(12):3169. doi: 10.3390/biomedicines10123169 (PMC9775971; doi:10.3390/biomedicines10123169)
Supplement: Supplementary file 1 [file biomedicines-10-03169-s001.zip › biomedicines-2030363-supplementary.pdf]

Supplementary Table S1. Results of blood cell count run on ABX Micros 60 at 3 concentration levels.

Serial	Medical laboratory	Blood cell concentration								
		HH1			HH2			HH3		
		Pseudo - leucocyts (10^9/L)	Human RBCs (10^{12}/L	Pseudo - platelets (10^9/L)	Pseudo - leucocyts (10^9/L)	Human RBCs (10^{12}/L	Pseudo - platelets (10^9/L)	Pseudo - leucocyts (10^9/L)	Human RBCs (10^{12}/L	Pseudo - platelets (10^9/L)
1	Medical Center District 7	3.60	3.15	94	8.40	4.53	152	27.50	5.70	419
2	Medical Center District 6	3.90	3.20	91	8.00	4.40	151	26.80	5.54	413
3	Binh Thanh District Medical Center	3.50	3.39	90	8.30	4.40	149	26.00	5.50	410
4	Ben Tre Traditional Medicine Hospital	3.65	3.56	97	8.20	4.48	159	26.60	5.70	409
5	Medical Center District 4	4.00	3.42	93	8.40	4.48	156	27.00	5.53	421
6	Medical Center District 5	4.40	3.46	97	8.40	4.45	154	27.00	5.76	420
7	Go Vap District Medical Center	3.60	3.15	99	8.00	4.30	145	26.50	5.90	419
8	Medical Center District 1	3.90	3.50	98	8.20	4.53	143	26.80	5.77	411
9	Medical Center District 10	4.10	3.51	91	8.30	4.44	158	26.90	5.70	424
10	Medical Center District 3	3.80	3.55	94	7.80	4.50	150	25.50	5.96	414

Supplementary Table S2. Results of blood cell count run on Celldyn 1700 at 3 concentration levels.

Serial	Medical laboratory	Blood cell concentration								
		HH1			HH2			HH3		
		Pseudo - leucocyts (10^9/L)	Human RBCs (10^{12}/L	Pseudo - platelets (10^9/L)	Pseudo - leucocyts (10^9/L)	Human RBCs (10^{12}/L	Pseudo - platelets (10^9/L)	Pseudo - leucocyts (10^9/L)	Human RBCs (10^{12}/L	Pseudo - platelets (10^9/L)
1	An Phu International Clinic	4.10	3.55	58	8.80	5.40	142	26.30	5.64	411
2	Van Phuoc General Clinic	4.00	3.63	57	8.80	5.07	143	26.50	5.18	410
3	Phong Tam Phuc General Clinic	4.01	3.61	58	814	5.57	147	26.72	5.86	415
4	Thien Tam 2 General Clinic	4.30	3.63	55	8.30	5.68	140	26.90	5.76	416
5	Sai Gon ITO Hung Dao General Clinic	4.60	3.57	53	9.50	5.53	135	29.70	5.70	424
6	Dong Nai Children's Hospital	4.50	3.58	59	8.00	5.05	139	25.00	5.00	420
7	Hoc Mon Area General Hospital	4.40	3.63	64	9.10	5.85	149	26.40	5.84	415
8	Thai Hoa General Clinic	4.70	3.60	60	8.10	4.90	142	26.00	6.00	418

9	Hoan Hao General Clinic	4.10	3.93	66	8.60	6.07	151	27.60	5.54	413
10	Victoria Healthcare My My Polyclinic	4.30	3.61	61	8.60	5.42	145	27.70	5.65	419

Supplementary Table S3. Results of blood cell count run on Mindray BC2000 at 3 concentration levels.

**Serial**	**Medical laboratory**	**Blood cell concentration**								
		**HH1**			**HH2**			**HH3**		
		**Pseudo - leucocyts (10^9/L)**	**Human RBCs (10^{12}/L**	**Pseudo - platelets (10^9/L)**	**Pseudo - leucocyts (10^9/L)**	**Human RBCs (10^{12}/L**	**Pseudo - platelets (10^9/L)**	**Pseudo - leucocyts (10^9/L)**	**Human RBCs (10^{12}/L**	**Pseudo - platelets (10^9/L)**
1	District 8 Hospital	3.60	3.00	64	7.20	5.20	140	24.00	5.30	395
2	410 Hoa Hao General Clinic	3.70	3.41	65	7.90	5.23	143	23.80	4.34	393
3	District 3 Hospital	3.90	3.53	63	8.00	5.24	135	24.90	5.73	392
4	District 4 Hospital	3.90	3.45	64	8.50	5.47	139	26.10	5.77	396
5	Cao Thang Eye Hospital	3.90	3.45	62	8.30	5.38	138	25.10	5.63	399
6	Saigon Plastic Surgery Specialist Hospital	3.80	3.40	61	7.30	5.10	135	24.60	5.40	390
7	Police Hospital HCMC	3.90	3.50	60	8.10	5.47	137	25.20	5.65	400
8	MAYO General Clinic	3.80	3.40	64	7.70	5.29	139	24.20	5.64	396
9	Can Tho Ear Nose Throat Hospital	4.01	3.69	61	8.20	5.57	136	25.00	5.80	390
10	The Gioi General Clinic	3.80	3.30	62	7.70	5.00	142	25.00	5.10	402
